# A switch from constitutive chemical defence to inducible innate immune responses in the invasive ladybird *Harmonia axyridis*

**DOI:** 10.1098/rsbl.2013.0006

**Published:** 2013-06-23

**Authors:** Henrike Schmidtberg, Christian Röhrich, Heiko Vogel, Andreas Vilcinskas

**Affiliations:** 1 Department Bioresources, Fraunhofer Institute for Molecular Biology and Applied Ecology (IME), Winchesterstrasse 2, 35394 Giessen, Germany; 2 Entomology Department, Max-Planck Institute for Chemical Ecology, Hans-Knoell Strasse 8, 07749 Jena, Germany; 3 Institute for Phytopathology and Applied Entomology, Justus Liebig University of Giessen, Heinrich Buff Ring 26–32, 35392 Giessen, Germany

**Keywords:** *Harmonia axyridis*, harmonine, insect immunity, invasion biology

## Abstract

The harlequin ladybird, *Harmonia axyridis*, has emerged as a model species for invasion biology, reflecting its remarkable capacity to outcompete native ladybird species when introduced into new habitats. This ability may be associated with its prominent resistance to pathogens and intraguild predation. We recently showed that the constitutive antibacterial activity present in the haemolymph of *H. axyridis* beetles can be attributed to the chemical defence compound harmonine. Here, we demonstrate that *H. axyridis* differs from other insects, including the native ladybird *Coccinella septempunctata,* by reducing rather than increasing the antimicrobial activity of its haemolymph following the injection of bacteria. However, both species produce new or more abundant proteins in the haemolymph, indicating that bacterial challenge induces innate immune responses associated with the synthesis of immunity-related proteins. Our results suggest that *H. axyridis* beetles can switch from constitutive chemical defence to inducible innate immune responses, supporting hypothesis that inducible antimicrobial peptides protect host beetles against pathogens that survive constitutive defences. These alternative antimicrobial defence mechanisms may reflect a trade-off resulting from fitness-related costs associated with the simultaneous synthesis of harmonine and antimicrobial peptides/proteins.

## 1. Introduction

The harlequin ladybird, multicoloured ladybird or Asian ladybird beetle (*Harmonia axyridis*) is a model species for invasion biology, because it can successfully outperform native ladybird species when introduced into new habitats [1]. This invasive success may be associated with its prominent resistance against pathogens [2] and intraguild predation [3]. We recently reported that the constitutive antimicrobial activity present in the haemolymph of this species is mediated by the chemical defence compound harmonine, also known as (17R,9Z)-1,17-diaminooctadec-9-ene, which displays broad-spectrum activity against even human pathogens, including the agents responsible for tuberculosis and malaria [4].

When we tested native ladybird species such as *Coccinella septempunctata*, we found that their haemolymph lacked the potent and constitutive antimicrobial activity found in *H. axyridis*, but that antimicrobial activity was induced by the injection of bacteria. Unexpectedly, we observed the opposite phenomenon in *H. axyridis*, i.e. constitutive antibacterial activity was inhibited following the injection of bacteria. These contrasting results led us to postulate that the injection of bacteria into *H. axyridis* causes a switch from the constitutive production of harmonine to the inducible synthesis of immunity-related proteins such as antimicrobial peptides. To address this hypothesis, we quantified antimicrobial activity and harmonine levels in untreated *H. axyridis* eggs, larvae and beetles, and beetles injected with a bacterial suspension or with a control buffer, and we investigated the haemolymph protein content of treated and untreated *H. axyridis* and *C. septempunctata* beetles by polyacrylamide gel electrophoresis. In addition, we used mass spectrometry to characterize the secretion of induced antimicrobial peptides into the haemolymph.

## 2. Material and methods

### (a) Injection of bacteria into beetles

*Harmonia axyridis* adults used for captive breeding were collected in and around Giessen and Ober-Moerlen, Germany, whereas *C. septempunctata* adults were obtained from Katz Biotech AG (Baruth, Germany). All ladybird species were reared in cages at 26°C and 60 per cent relative humidity, with a 16 h photoperiod. Bean plants (*Phaseolus vulgaris*) infested with pea aphids (*Acyrthosiphon pisum*) were provided as a food source. We injected 10 µl of bacterial suspension diluted with anti-coagulant saline to 4.75 × 10^8^ cfu containing *Micrococcus luteus* DSM 20030 and *Escherichia coli* D31 into each adult *H. axyridis* and *C. septempunctata* beetle, using a Nanolitre 2000 microinjector and a Sy-Micro4 controller (World Precision Instruments). Control beetles were injected with 10 µl anti-coagulant saline (69 mM KCl, 27 mM NaCl, 2 mM NaHCO_3_, 100 mM d(+)-glucose, 30 mM tripotassium citrate, 26 mM citric acid, 10 mM Na_2_-EDTA, pH 4.6). After 24 h, the immune-challenged specimens and controls were fixed to a tape under a stereomicroscope, and haemolymph samples were taken by cutting the legs at their coxal base and drawing the haemolymph (5–10 µl per beetle) into ice-cold anti-coagulant saline (final dilution 1 : 2). Haemolymph was also taken from untreated and in mock-injected control adult beetles and larvae of both species.

### (b) Antimicrobial activity assay

The antimicrobial activity of the haemolymph was determined using the common agar diffusion assay on *E. coli* D31 and *M. luteus* DSM 20030 plates as described previously [4]. Wells (3 mm diameter) were punched from the agar plates, and loaded with 3 µl of the haemolymph samples from untreated larvae/beetles and challenged beetles of each ladybird species. Eggs from both species were squeezed directly onto the agar plates. After incubation at 37°C for 24 h, the diameters of the inhibition zones were recorded. Different concentrations of gentamycin sulfate (Roth) and lysozyme (Fluka) were used as standards for calibration. The antibacterial activity of the haemolymph was presented as µg ml^−1^ gentamycin sulfate or lysozyme equivalents. For statistical calculations, one-way ANOVA and the Holm–Sidak test were performed by using SigmaPlot. A *p* < 0.05 was considered as statistically significant (see the electronic supplementary material).

### (c) Gel electrophoresis and LC-MS^E^ identification of haemolymph proteins

Haemolymph samples were fractionated by sodium dodecylsulfate polyacrylamide gel electrophoresis (SDS-PAGE) in 16.5 per cent tricine gradient gels (Bio-Rad), to determine the effect of bacterial challenge on haemolymph protein composition. The haemolymph from 10 adult beetles of each species (untreated and challenged) was collected into ice-cold phosphate-buffered saline (final dilution 1 : 2), centrifuged at 1500 rcf for 10 min at 4°C, and the supernatant was stored at –20°C. The separated proteins were stained using EZBlue (Sigma) and the bands were analysed using Quantity One 1-D analysis software (Bio-Rad). Lane comparison data were plotted with SigmaPlot v. 8.0. Bio-Rad polypeptide molecular-weight standards were used as size markers. The identification of the induced, low-molecular-weight protein bands by liquid chromatography mass spectrometry in elevated energy mode (LC-MS^E^) has been described previously [5].

### (d) Determination of harmonine concentration by mass spectroscopy

Before haemolymph was collected, the mass of individual *H. axyridis* eggs, larvae and adults was determined. Each individual was then squeezed in 0.2 ml 20 per cent acetonitrile, vortexed for 30 s, ultrasonicated for 30 min and centrifuged for 30 min at 21 000 rcf at 4°C to remove insoluble material. A defined volume of the supernatant was transferred to a HPLC glass vial and diluted 1 : 200 with 20 per cent acetonitrile before injection. Harmonine was separated using an UltiMate 3000 HPLC (Dionex) with a gradient of 20–60% acetonitrile in 10 min and a flow-rate of 250 µl/min on a reversed-phase column (Acclaim 120, C_18_, 3 µm, 2.1 × 150 mm; Dionex) at 20°C, and quantified using a micrOTOF-Q II mass spectrometer (Bruker Daltonics) with an orthogonal electrospray ionization source. The main signal of the extract ion chromatogram (*m/z* = 283,3 ± 0.2 Da) was integrated and correlated with the signal of synthetic harmonine.

## 3. Results

### (a) Antimicrobial activity in ladybird beetle haemolymph

**Figure 1.**
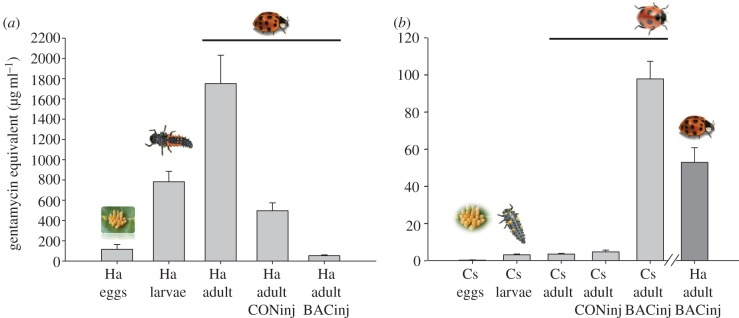
The antimicrobial activity of eggs and haemolymph against *Escherichia coli* D31 was tested using an agar diffusion assay. (*a*) The antimicrobial activity of untreated *Harmonia axyridis* (Ha) eggs, final-instar larvae and beetles increased throughout development, whereas the antimicrobial activity significantly declined in adults injected with buffer (Ha adult CONinj) or bacteria (Ha adult BACinj). (*b*) In *Coccinella septempunctata* (Cs), the antibacterial activity in untreated eggs, larvae and adults, as well as beetles injected with buffer alone (Cs adult CONinj) was low compared with that observed in beetles following injection with bacteria (Cs adult BACinj). The antibacterial activity of bacteria injected adult *H. axyridis* (dark grey) is almost comparable with the activity of bacteria injected *C. sepempunctata.* The results are mean values ± s.e. calculated from independent experiments (further details and the results of corresponding experiments using *M. luteus* as an indicator organism are provided in the electronic supplementary material).

We tested the haemolymph of untreated *C. septempunctata* beetles and found no activity against either *E. coli* or *M. luteus*, whereas the eggs, larvae and adults of *H. axyridis* displayed increasing activity as demonstrated by the successively larger inhibition zones (figure 1 and table 1).

**Table 1.** Comparative statistical significance analysis of antimicrobial activity against *E. coli* in *C. septempunctata* (Cs) and *H. axyridis* (Ha). (Holm–Sidak test with overall significance level of 0.05 (bold).)

	larvae	adult	adult CONinj	adult BACinj
groups (Cs)				
Cs eggs	0.760	0.719	0.642	**<0**.**001**
Cs larvae		0.955	0.837	**<0.001**
Cs adult			0.872	**<0.001**
Cs adult CONinj				**<0.001**
groups (Ha)				
Ha eggs	0.087	**<0**.**001**	0.350	0.861
Ha larvae		**0.002**	0.456	**0.031**
Ha adult			**<0.001**	**<0.001**
Ha adult CONinj				0.216

The injection of a bacterial suspension into *C. septempunctata* beetles strongly induced antibacterial activity in the haemolymph, resulting in a clear inhibition zone that was not observed with the non-injected beetles (figure 1 and table 1). Surprisingly, the injection of bacterial suspensions into *H. axyridis* beetles caused the constitutive antibacterial activity to decline considerably. Based on the size of the inhibition zones, bacterial challenge resulted in both ladybird species displaying comparable levels of antibacterial activity in the haemolymph.

### (b) Detection of harmonine by mass spectrometry

We measured the levels of harmonine relative to body mass (µg mg^−1^) in *H. axyridis* and found that the quantities during development and in the challenge experiments correlated precisely with the zones observed in the inhibition assays (see the electronic supplementary material, figure S2). The levels of harmonine increased during development to a high level in adult beetles (see the electronic supplementary material, figure S1), but declined following bacterial challenge. We used both synthetic and purified harmonine to confirm that the observed strong antimicrobial activity in *H. axyridis* control eggs, larvae and adults could be attributed directly to this defence alkaloid.

### (c) Changes in the haemolymph proteome

**Figure 2.**
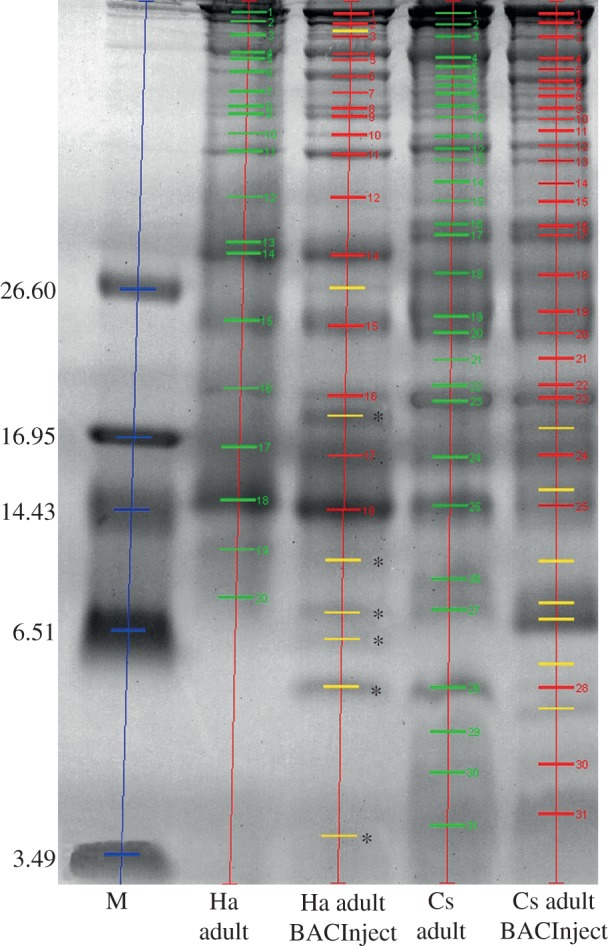
The protein composition of haemolymph from untreated *H. axyridis* (Ha adult) and *C. septempunctata* (Cs adult) beetles, and the same beetles injected with bacteria (Ha adult BACInject, Cs adult BACInject) was investigated by SDS-PAGE. Green bands represent proteins in haemolymph samples from untreated specimens. Matching protein bands with corresponding molecular weights identified in the haemolymph of immune-challenged animals are shown in red. Yellow bands appear only after the immune challenge. Lane 1 contains marker proteins (M) with molecular weights (kDa) shown on the left. Bands used for protein identification by LC-MS^E^ are depicted by asterisks.

The haemolymph contains a relatively small number of proteins and peptides, which makes it suitable for analysis by one-dimensional SDS-PAGE. We, therefore, compared the banding patterns of fractionated haemolymph samples from *H. axyridis* and *C. septempunctata* beetles 24 h after injection with the bacterial suspension, and from untreated controls. We observed a number of qualitative and quantitative differences between the post-injection and control samples, with several more prominent bands in the post-injection sample as well as novel bands potentially representing immunity-related proteins (figure 2). The most significant changes were observed in the low-molecular-mass range of the gel, consistent with the characteristics of antimicrobial peptides. Using our previously established *H. axyridis* transcriptomic database [6], we analysed six protein bands by LC-MS^E^ (figure 2). We positively identified eight antimicrobial peptides, including defensins (Def1, Def9, SapL8), coleoptericins (Col2, Col6, Col8) and coleoptericin-like peptides (ColLB, ColLC).

## 4. Discussion

We screened the haemolymph of the native ladybird *C. septempunctata* and the invasive ladybird *H. axyridis* for antibacterial activity using a standard *E. coli* and *M. luteus* inhibition assay. We found that constitutive antibacterial activity was present only in *H. axyridis* [4], but could be induced in the non-invasive species *C. septempunctata* by the injection of a bacterial suspension, which is consistent with observations in other insects [5]. Remarkably, the injection of bacteria into *H. axyridis* caused a considerable decline in the antibacterial activity of the haemolymph, as shown by the smaller post-injection inhibition zone.

The antibacterial activity detected in the eggs, larvae and adults of *H. axyridis* correlated with the amounts of harmonine in the haemolymph, as determined by mass spectrometry (see the electronic supplementary material, figures S1 and S2). The highest harmonine concentrations were found in the haemolymph of untreated adult beetles (up to 7 µg mg^−1^), but the loss of antibacterial activity following bacterial injection into adult beetles reflected a considerable decline in harmonine levels. These data suggest that harmonine is primarily responsible for the observed constitutive antimicrobial activity of the haemolymph, although it is unclear whether the compound is degraded or modified when the immune system is challenged with the bacterial suspension. However, the remaining antimicrobial activity detected in *H. axyridis* after the injection of bacteria was comparable with that found in the haemolymph of *C. septempunctata* beetles treated in the same manner. Our mass spectrometry data and harmonine activity assays confirmed that the antimicrobial activity in the haemolymph of immune-challenged larvae cannot be attributed to the harmonine content.

Instead, we proposed that the antibacterial activity in the *H. axyridis* haemolymph following bacterial challenge is mediated by antimicrobial peptides that compensate for the reduced levels of harmonine. This is supported both by the loss of harmonine from the *H. axyridis* haemolymph following injection (see the electronic supplementary material, figure S1) and the increased abundance and diversity of haemolymph proteins in both ladybird species, representing secreted immunity-related proteins and peptides (figure 2). To test this hypothesis, we analysed the induced low-molecular-mass bands by mass spectrometry using the LC-MS^E^ method in combination with our recently published *H. axyridis* transcriptome database [6]. We identified eight antimicrobial peptides, including defensins and coleoptericins, confirming the induction of an innate immune response.

Our data show that *H. axyridis* can switch from constitutive chemical antimicrobial defence (based on harmonine) to innate immune responses (based on antimicrobial peptides). The resources that can be used to synthesize either harmonine or antimicrobial peptides are limited, so *H. axyridis* provides an excellent model for investigation potential trade-offs resulting from investments into different defence mechanisms associated with corresponding fitness components, a virtual currency that combines survival with reproduction [7,8]. Our data also support the hypothesis that inducible effector molecules are produced in order to protect host beetles against pathogens that survive an initial barrier of constitutive defences [9,10].
